# Impairing photorespiration increases photosynthetic conversion of CO_2_ to isoprene in engineered cyanobacteria

**DOI:** 10.1186/s40643-021-00398-y

**Published:** 2021-05-21

**Authors:** Jie Zhou, Fan Yang, Fuliang Zhang, Hengkai Meng, Yanping Zhang, Yin Li

**Affiliations:** 1 CAS Key Laboratory of Microbial Physiological and Metabolic Engineering, State Key Laboratory of Microbial Resources, Institute of Microbiology, Chinese Academy of Sciences, No. 1 West Beichen Road, Chaoyang District, Beijing, 100101 China; 2 University of Chinese Academy of Sciences, Beijing, China; 3 School of Life Sciences, University of Science and Technology of China, Hefei, China; 4 CAS Key Laboratory of Microbial Physiological and Metabolic Engineering, State Key Laboratory of Transducer Technology, Institute of Microbiology, Chinese Academy of Sciences, Beijing, China

**Keywords:** Impairing photorespiration, Photosynthetic conversion of CO_2_, Isoprene production, Glycolate dehydrogenase, Cyanobacteria

## Abstract

Photorespiration consumes fixed carbon and energy generated from photosynthesis to recycle glycolate and dissipate excess energy. The aim of this study was to investigate whether we can use the energy that is otherwise consumed by photorespiration to improve the production of chemicals which requires energy input. To this end, we designed and introduced an isoprene synthetic pathway, which requires ATP and NADPH input, into the cyanobacterium *Synechocystis* sp. 6803. We then deleted the *glcD1* and *glcD2* genes which encode glycolate dehydrogenase to impair photorespiration in isoprene-producing strain of *Synechocystis*. Production of isoprene in *glcD1/glcD2* disrupted strain doubled, and stoichiometric analysis indicated that the energy saved from the impaired photorespiration was redirected to increase production of isoprene. Thus, we demonstrate we can use the energy consumed by photorespiration of cyanobacteria to increase the energy-dependent production of chemicals from CO_2_.

**Supplementary Information:**

The online version contains supplementary material available at 10.1186/s40643-021-00398-y.

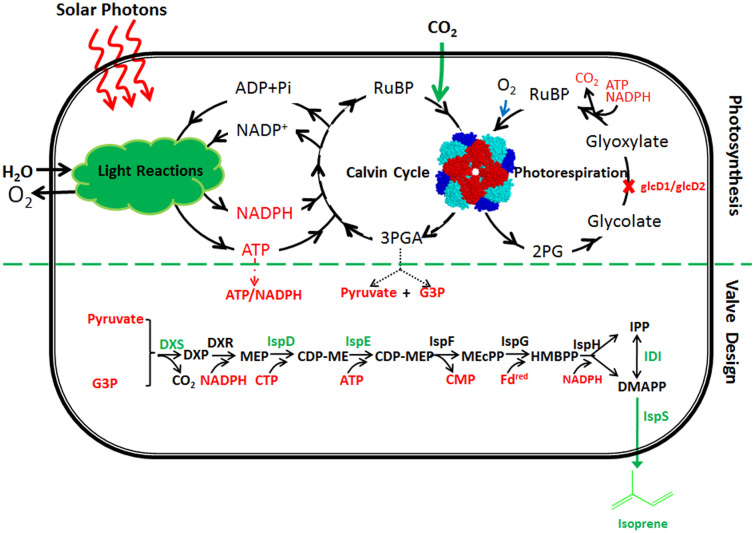

## Introduction

Photorespiration refers to the metabolism of 2-phosphoglycolate (2-PG), which is derived from the oxygenase activity of ribulose-1,5-bisphosphate carboxylase/oxygenase (Rubisco) (Tolbert 1997). Usually one-third to one-fourth of the generated ribulose-1,5-bisphosphate (RuBP) is channeled into photorespiration in higher plants (Hagemann and Bauwe 2016). The energy consumed by photorespiration accounts for up to one-third of the total energetic cost of CO_2_ fixation (Hagemann and Bauwe 2016). Avoiding photorespiration is, therefore, a key target for improving crop yields and effort for removing or reducing photorespiration has never been stopped.

If the oxygenase activity of Rubisco can be deactivated, the photorespiration could be removed, as there will be no 2-PG available. To increase photosynthetic efficiency, engineering Rubisco, including increasing the carboxylase activity and/or decreasing the oxygenase activity of Rubisco, has been one of the holy grails of photosynthetic research. However, due to the extremely complex structure of Rubisco, little improvement was achieved (Cai et al. 2014; Shih et al. 2014).

Since photorespiration cannot be removed from deactivation of the oxygenase activity of Rubisco, impairing the metabolic pathway of 2-PG might prevent loss of the fixed carbon and energy from the photorespiratory 2-PG cycle. However, impairing photorespiratory 2-PG cycle in higher plants (Kozaki and Takeba 1996; Engel et al. 2007) or in cyanobacteria (Eisenhut et al. 2008) all resulted in high-CO_2_ requirement for normal growth, which is termed as the high-CO_2_-requiring (HCR) phenotype. The HCR phenotype means cells could not grow under ambient conditions and require more CO_2_ or low light for growth. To date, effort for increasing photosynthesis efficiency through impairing the photorespiration has been unsuccessful (Hagemann and Bauwe 2016).

Engineering photorespiration into photorespiratory bypass might partially reduce the loss of fixed carbon and photochemical energy consumed by the photorespiratory 2-PG cycle. In 2007, a bacterial glycolate catabolic pathway was introduced into *Arabidopsis thaliana* chloroplasts. Part of the photorespiratory glycolate was converted into CO_2_ and glycerate, the latter was then channeled into the Calvin–Benson–Bassham cycle (Kebeish et al. 2007). A reduced photorespiration and an increased biomass production were observed under ambient conditions (Kebeish et al. 2007). In 2014, a synthetic CO_2_-fixing photorespiratory bypass was introduced into cyanobacterium. However, no significant improvement on photosynthesis was observed (Shih et al. 2014). Recently, introducing alternative glycolate metabolic pathways into tobacco chloroplasts resulted in a more than 40% increased biomass productivity (South et al. 2019; Fernie and Bauwe 2020).

It has long been considered that photorespiration is superfluous or incomplete in cyanobacteria because of their CO_2_-concentrating mechanism (CCM) (Colman 1989; Eisenhut et al. 2008). However, it was shown recently that photorespiration is essential in cyanobacteria (Eisenhut et al. 2019). Photorespiration-impaired cyanobacterium strain required high CO_2_ for normal growth and 2-phosphoglycolate phosphatases, a key enzyme involved in photorespiration, were identified in *Synechocystis* sp. PCC 6803 (hereafter termed *Synechocystis*), indicating photorespiration is essential in cyanobacteria (Eisenhut et al. 2008; Dyo and Purton 2018). Since recently, cyanobacteria has been widely engineered for production of chemicals from CO_2_, and some chemicals are derived from pathways that require strong input of energy and reducing equivalent, like isoprene. We therefore wonder if we could impair the photorespiration of cyanobacteria, can we use the energy that is otherwise consumed by photorespiration to improve the production of chemicals whose synthetic pathway is dependent on energy supply? To this end, we chose cyanobacterium *Synechocystis* as a model strain, to which an isoprene biosynthesis pathway was introduced and optimized. We then impaired the photorespiration and investigate the consequence.

## Materials and methods

### Construction of plasmids

All plasmids and primers are listed in Additional file 1: Tables S1 and S2. The plasmid pSM2-ispS was constructed by inserting the P_cpc560_-ispS expression cassette, which was optimized and synthesized by GENEWIZ. Inc. (China), into plasmid pSM2 (Zhou et al. 2012, 2016). Plasmid pSM1-MEP* was constructed by inserting the Pcpc-*dxs-idi-isp*D-*isp*F expression cassette, which was synthesized by GENEWIZ. Inc. (China), into pSM1 (Zhou et al. 2012, 2016).

The plasmid pEasy-ΔglcD1 was constructed by inserting the *glc*D1 knockout cassette into pEasy-blunt-simple. The *glc*D1 knockout cassette comprised a sequence complementary to the 700 bp upstream of *glc*D1 (*glc*D1 up), the erythromycin resistance cassette Em^r^ and a sequence complementary to the 700 bp downstream of *glc*D1 (*glc*D1 down). pEasy-ΔglcD2 was constructed by inserting the *glc*D2 knockout cassette into pEasy-blunt-simple. The *glc*D2 knockout cassette comprised a sequence complementary to the 600 bp upstream of *glc*D2 (*glc*D2 up), a spectinomycin resistance cassette and a sequence complementary to the 600 bp downstream of *glc*D2 (*glc*D2 down).

### Construction of *Synechocystis* mutant strains and growth conditions

All strains are listed in Additional file 1: Table S1. Transformation of *Synechocystis* using the constructed plasmids listed in Additional file 1: Table S1 was performed as described previously (Lindberg et al. 2010a). All primers used in this work are listed in Additional file 1: Table S2.

The growth conditions for all *Synechocystis* strains were the same as described previously (Zhou et al. 2016), with the exception of supplementing 50 mM NaHCO_3_ which was necessary for the growth of the photorespiration-impaired (HCR) strain ΔglcD1/D2. Chloromycetin (10 μg mL^−1^) and/or kanamycin (10 μg mL^−1^) and/or erythromycin (30 μg mL^−1^) and/or spectinomycine (10 μg mL^−1^) were added to the medium, when necessary.

### Isoprene production assay

To measure isoprene production, the IspS, MEP*-IspS, IspSΔglcD1/D2 and MEP*-IspSΔglcD1/D2 mutants were grown in 140 mL sealed bottles containing 70 mL of BG11 medium with 50 mM NaHCO_3_ under a constant illumination intensity of 100 μmol photons m^−2^ s^−1^. Cell growth and isoprene production were monitored every day by measuring the OD at 730 nm and headspace gas analysis via gas chromatography, respectively.

1 mL gas samples from the headspace of sealed bottles was collected using an airtight syringe and were analyzed on a GC-2014 gas chromatograph (Shimadzu, Kyoto, Japan) equipped with a flame ionization detector and an HP-AL/S column (15 m, 0.53 mm inside diameter, 0.15 μm film thickness; Agilent Technologies, Santa Clara, CA, USA) using nitrogen as carried gas. Vaporized pure isoprene was used as the standard. The starting temperature of the oven was 40 °C and was maintained for 5 min as described previously (Bentley and Melis 2012).

### Quantification of extracellular and intracellular glycolate concentration

All strains were grown in 250 mL flasks containing 50 mL of BG11 medium supplemented with 50 mM NaHCO_3_ for 4 days to OD_730_ of approximately 3.0, under a constant illumination intensity of 100 μmol photons m^−2^ s^−1^. Extracellular glycolate concentrations were determined using the culture supernatants. To prepare samples for intracellular glycolate concentration, cell pellets were collected by vacuum filtration using a nylon membrane filter (0.44 μm, 50 mm). Each filter was transferred to a 50 mL centrifuge tube and the extraction was carried out with 5 mL 80% ethanol at 65 °C for 3 h. After centrifugation, the supernatants were collected and dried by lyophilization and subsequently redissolved in 500 μL of water (Eisenhut et al. 2006, 2008).

To quantify the extracellular glycolate or intracellular glycolate, 1 μL of culture supernatant or 1 μL of the redissolved cell extraction was analyzed by HPLC equipped with Bio-Rad Aminex® HPX-87H Ion Exclusion Column (300 mm × 7.8 mm) using 8 mM H_2_SO_4_ as mobile phase, pumped at a flow rate of 0.6 mL min^−1^. The column temperature was maintained at 50 °C, peaks were detected using Agilent Technologies 1260 RID (refractive index detector) (Eisenhut et al. 2006).

### Quantification of extracellular ^13^C-labeled glycolate concentration by LC–MS

To follow the fate of glycolate, 1 mM ^13^C-labeled glycolic acid (1,2-13C2; 99% pure; Cambridge Isotope Lab) was added to the shake flask cultures after grown for 2 days under the conditions described previously. Subsequently, the cells were cultivated for 2 days, the extracellular concentration of ^13^C-labeled glycolate were analyzed by LC–MS.

100 μL aliquots of culture supernatants were mixed with 900 μL methanol and centrifuged for 5 min at 20,000×*g*. 500 μL aliquots of supernatants were lyophilized and sequentially resuspended in 500 μL of water.

The extracellular concentrations of ^13^C-labeled glycolate were analyzed using an ExionLC HPLC system (AB SCIEX) coupled with a 6500 Q-TRAP mass spectrometer (AB SCIEX). Glycolate were separated with a HyperREZ XP Organic acid column (10 cm length, 7.7 mm diameter, 8 µm particle size; Thermo ScientificTM) using H_2_O as mobile phase. The column was maintained at 40 °C with a solvent flow rate of 0.4 mL min^−1^. Injection volume was 10 µL. Electrospray ionization was used in negative mode.

### Intracellular GAP and pyruvate assay

3-P-glycerate and pyruvate were extracted as the method described previously (Gao et al. 2016) and analyzed using the LC–MS methods as described above.

### Analysis of PSII chlorophyll fluorescence

Parameters of chlorophyll fluorescence kinetics, including the effective quantum yield (*Y*(II) = *F*_v_′/*F*_m_′) and the relative electron transport rate of PSII (rETR(II), were measured on a Dual-PAM-100 instrument (Walz, Germany) as described previously (Zhou et al. 2016).

### Measurement of the P700 signal

The redox state of the reaction center chlorophyll of PSI (P700) was determined on a Dual-PAM-100 instrument (Walz, Germany), as described previously (Zhou et al. 2016). The PSI effective quantum yield (*Y*(I) = *P*_v_′/*F*_m_′), the relative electron transport rate (rETR(I)) via PSI, and the PSI complementary quantum yields of non-photochemical energy dissipation, *Y*(ND) and *Y*(NA) were also analyzed (Zhou et al. 2016).

### Oxygen evolution

Measurements of the oxygen evolution rate of all strains were performed using an Oxygraph Plus Clark-type electrode (Hansatech, UK) as described previously (Zhou et al. 2016). The OD_730_ of all strains was set to 6.0 for the measurements.

### Stoichiometric analysis

Oxygenation of 1 mol Ribulose biphosphate (RuBP) results in the formation of 1 mol glycerate-3-phosphate (3-PGA) and 1 mol glycolate-2-phophate (2-PG), which is converted into 0.5 mol 3-PGA and releases 0.5 mol CO_2_ in the photorespiratory pathway. As described in a previous literature (Bauwe et al. 2010), cyanobacterial 2-PG metabolism can directly convert glyoxylate into hydroxypyruvate with glyoxylate carboligase and tartronic semialdehyde reductase. During this process, no NH_3_ was released and re-assimilated. Since the carbon fixation rate was not altered significantly, the re-fixation of CO_2_ released from photorespiration can be involved in the normal photosynthesis, and, thus, is not calculated in this study. As shown in Additional file 1: Table S1, 1 mol ATP will be required to convert 1 mol glycolate into glyceraldehyde-3-phosphate (G3P), while 54 mol ATP will be required for biosynthesis of 1 mol isoprene from CO_2_. The amounts of ATP and NAD(P)H consumed in isoprene biosynthesis and saved from blocking glycolate metabolism were calculated according to the titer of isoprene and glycolate determined at 96 h, respectively.

## Results and discussion

### Design and construction of an energy consuming isoprene synthetic pathway in *Synechocystis*

**Fig. 1:**
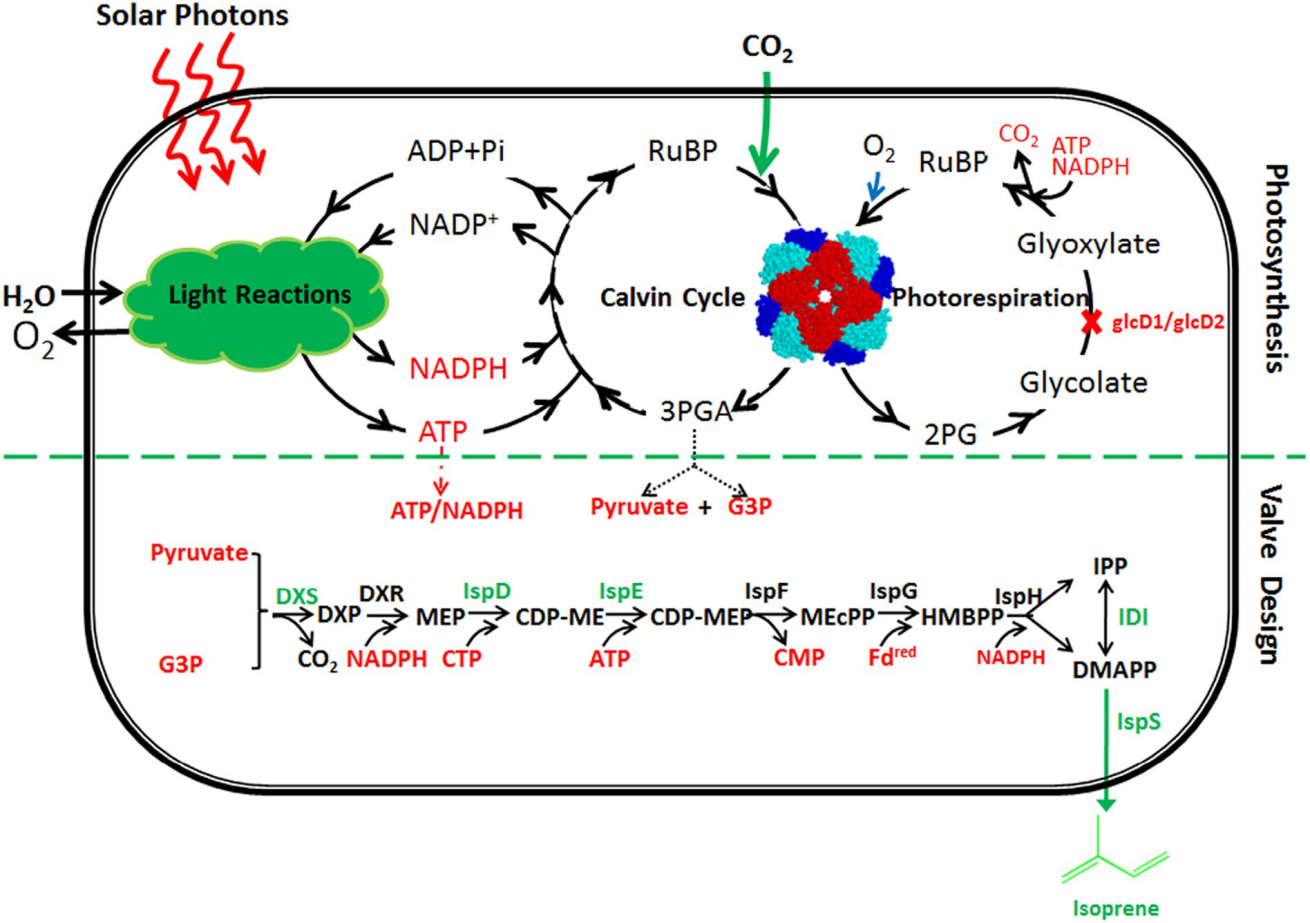
Design and creation of an isoprene synthetic pathway in *Synechocystis*. The isoprene synthetic pathway is a MEP-dependent and energy consuming pathway. An exogenous isoprene synthase (IspS, from *Pueraria montana*) was introduced to convert DMAPP to isoprene. To optimize the isoprene-synthetic pathway, *dxs*, *idi*, *ispD* and *ispF* from *E. coli* were overexpressed in *Synechocystis*. *RuBP* ribulose-1,5-bisphosphate, *3PGA* 3-phosphoglycerate, *2PG* 2-phosphoglycolate, *G3P* glyceraldehyde-3-phosphate, *DXP* 1-deoxy-d-xylulose-5-phosphate, *MEP* methylerythritol phosphate, *CDP-ME* diphosphocytidylyl methylerythritol, *CDP-MEP* diphosphocytidylyl methylerythritol 2-phosphate, *MEcPP* methylerythritol 2,4-cyclodiphosphate, *HMBPP* hydroxymethylbutenyl 4-diphosphate, *IPP* isopentenyl pyrophosphate, *DMAPP* dimethylallyl pyrophosphate. Enzymes: *DXS* DXP synthase, *DXR* DXP reductoisomerase, *IspD* 4-diphosphocytidyl-2C-methyl-d-erythritol synthase, *IspE* 4-(cytidine-5′-diphospho)-2-C-methyl-d-erythritol kinase, *IspF* 2C-methyl-d-erythritol 2,4-cyclodiphosphate synthase, *IspG* 4-hydroxy-3-methylbut-2-enyl-diphosphate synthase, *IspH* HMBPP reductase, *IDI* IPP isomerase, *IspS* isoprene synthase

In addition to detoxification of 2-PG and salvage of organic carbon (Bloom 2015), it is generally accepted photorespiration plays an important physiological function that is to protect the host from photoinhibition by dissipating excess photochemical energy (Kozaki and Takeba 1996). When the capacity of photorespiration in higher plant was improved or decreased, the tolerance to high-intensity light was increased or decreased, respectively (Kozaki and Takeba 1996). We, therefore, hypothesized if we could introduce a photochemical energy consuming pathway into cyanobacteria, we might be able to impair photorespiration without generating HCR phonotype, as the physiological function of photorespiration can be replaced by the introduced energy consuming pathway. In this way, the energy that is otherwise consumed in photorespiration can be saved and used to increase the production of target chemicals. We noticed that *Synechocystis* has a methylerythritol phosphate (MEP) pathway, which consumes 3 ATP and 3 NADPH (Fig. 1). If we could engineer this pathway to allow it produce isoprene from CO_2_, we might be able to test whether the above hypothesis would work.

**Fig. 2:**
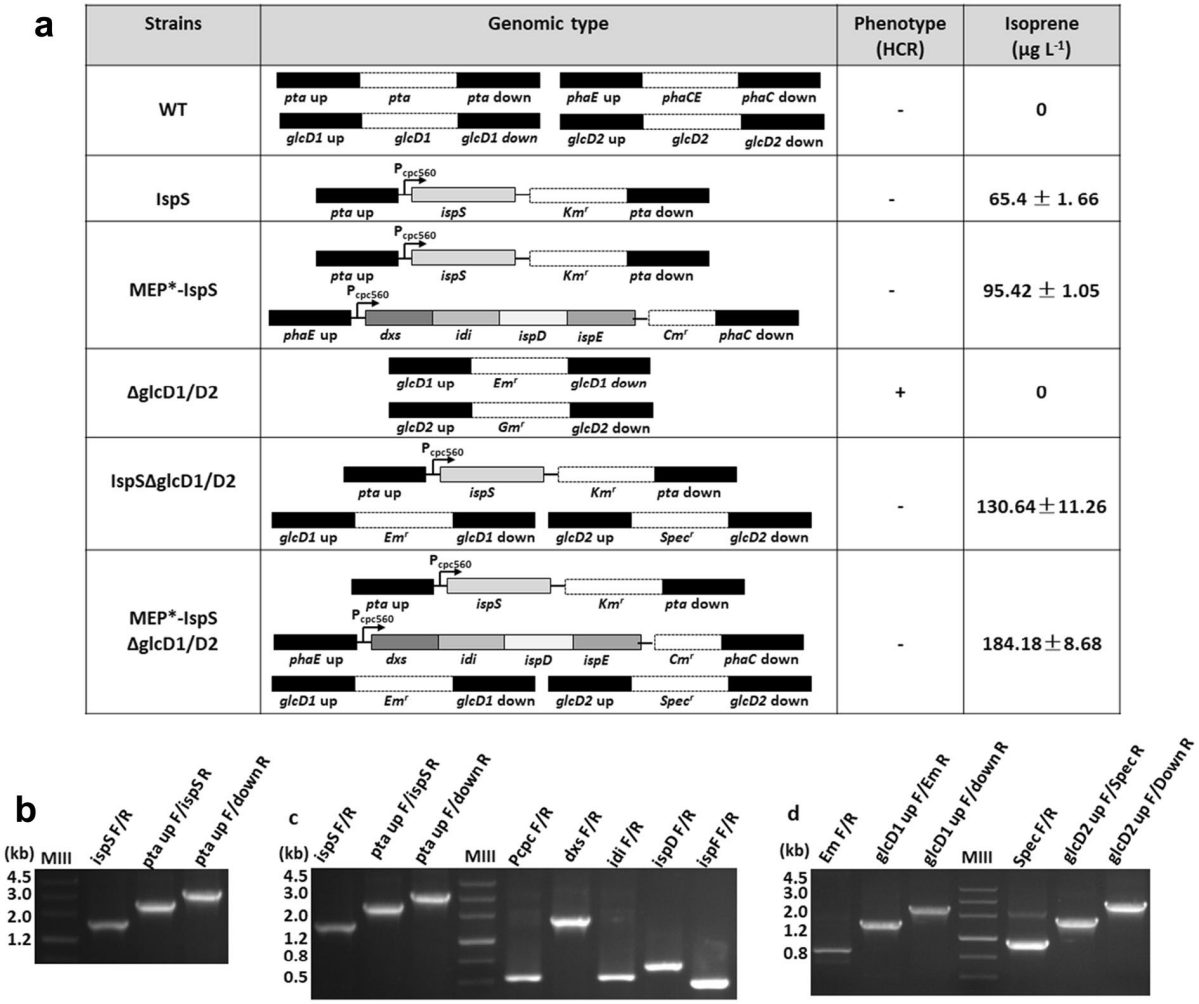
Construction of mutant strains. **a** Genetic modifications to impair photorespiration and introduce the isoprene synthetic pathway. **b** Whole-cell PCR for the recombinant cassette harboring the *ispS* gene at the *pta* site; one primer was set 100 bp external to the recombinant cassette, which demonstrated the complete segregation of the Isps mutant. **c** Whole-cell PCR with specific primers demonstrating the integration of each gene into the chromosome of the recombinant strain MEP*-IspS. **d** Whole-cell PCR with specific primers for complete segregation of knocking out *glcD1* and *glcD2* gens in photorespiration impairing mutantsΔglcD1/D2, IspSΔglcD1/D2 and MEP*-IspSΔglcD1/D2

To engineer the energy consuming MEP pathway into an isoprene synthetic bypass, we introduced the isoprene synthase encoding gene *ispS* from the higher plant kudzu (*Pueraria montana*) (Lindberg et al. 2010b) into *Synechocystis*, to catalyze the conversion of dimethylallyl diphosphate (DMAPP) into isoprene (Fig. 1). The codon-optimized *ispS* gene was placed under the control of the strong P_cpc560_ promoter (Zhou et al. 2014), and inserted into the *pta* site (Zhou et al. 2012) of *Synechocystis*, generating the first-generation isoprene-producing strain, designated as IspS (Fig. 2a).

Since the activity of the MEP pathway determines the consumption of photochemical energy, we further optimized the MEP pathway with the aim to generate a second generation isoprene-producing strain. Previous studies on engineering the MEP pathway for the production of isoprene or isoprenoid compounds in microorganisms have demonstrated that the intracellular concentration of DMAPP can be increased by increasing the expression levels of *dxs*, encoding 1-deoxy-d-xylulose-5-phosphate synthase, *idi*, encoding isopentenyl pyrophosphate (IPP) isomerase, *ispD*, encoding 4-diphosphocytidyl-2C-methyl-d-erythritol synthase, and *ispF*, encoding 2C-methyl-d-erythritol 2,4-cyclodiphosphate synthase (Gao et al. 2016; Ajikumar et al. 2010). We therefore cloned *dxs*, *idi*, *ispD,* and *ispF* from *E. coli* and assembled these genes into an artificial operon under the control of a strong promoter P_cpc560_ (Zhou et al. 2014), and inserted the artificial operon into the *phaCE* site of the IspS strain. The resulting second-generation isoprene-producing strain was designated as MEP*-IspS (Fig. 2a).

**Fig. 3:**
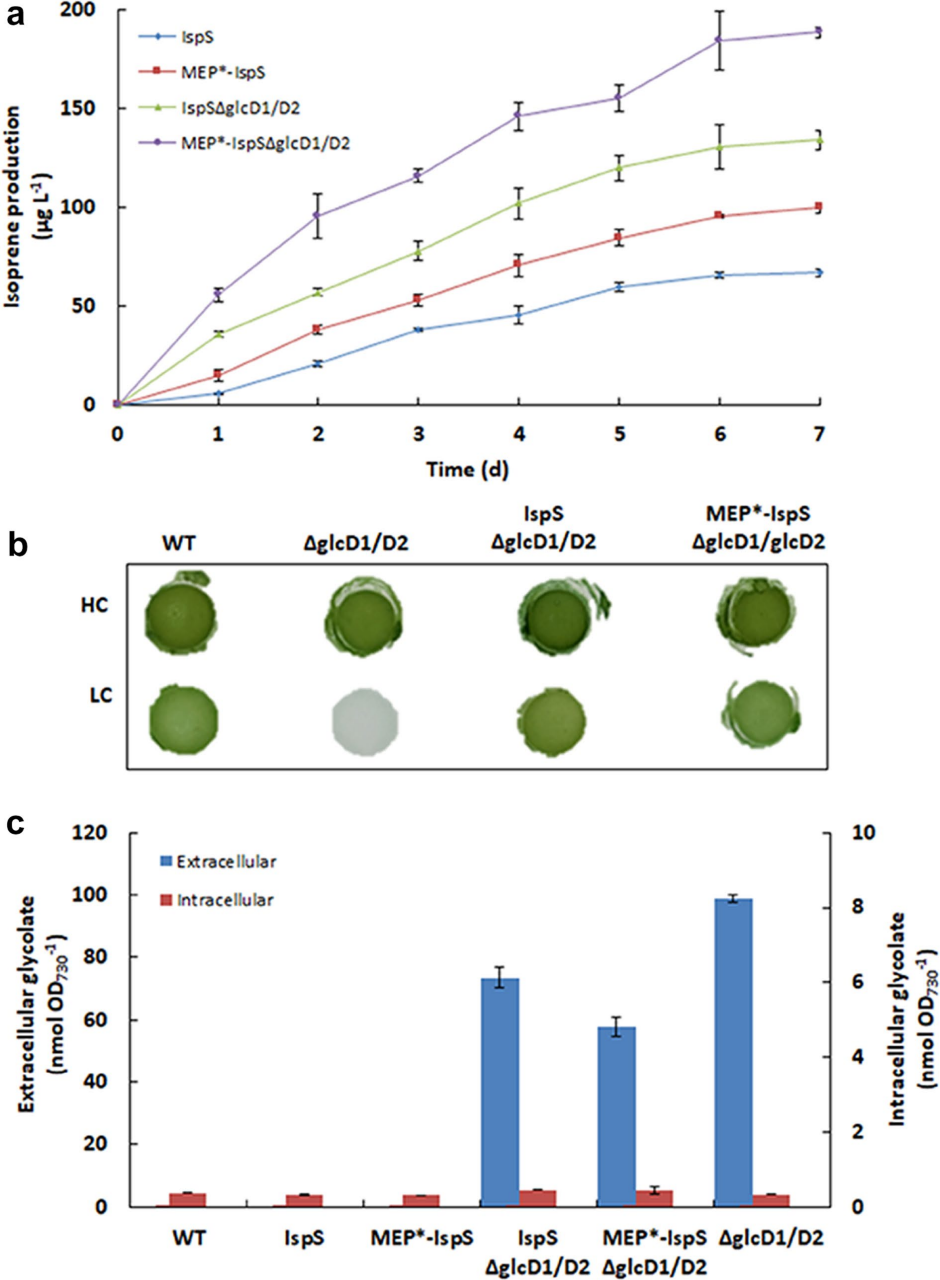
Phenotype analysis of all strains. **a** Time-course of isoprene productions by the strains IspS, MEP*-IspS, IspSΔglcD1/D2 and MEP*-IspSΔglcD1/D2. **b** High-CO_2_-requiring (HCR) phenotype analysis of the photorespiration-impaired strains ΔglcD1/D2, IspSΔglcD1/D2 and MEP*-IspSΔglcD1/D2. The strains were grown on BG11 plates with (HC) or without (LC) 50 mM NaHCO_3_ under 100 μmol photons m^−2^ s^−1^ light condition. **c** Quantification of intracellular and extracellular glycolate in WT and all mutant strains grown on HC condition

Complete segregation and correct gene insertion were verified by PCR and sequencing (Fig. 2b, c). Metabolite spectrum analysis indicated that the mutant IspS strain constructed in this work produced 65.4 ± 1.7 µg L^−1^ of isoprene when incubated in BG11 medium at 30 °C under constant white light for 6 days, while the mutant MEP*-IspS produced 95.4 ± 1.0 µg L^−1^ (Figs. 2a, 3a). These results confirmed that two mutants equipped with energy consuming MEP pathways of different activities were obtained.

### Impairing photorespiration increased isoprene production in IspS and MEP*-IspS strains

To test whether we can use the energy that is otherwise consumed by photorespiration to improve the photosynthetic conversion of CO_2_ to isoprene, we tried to impair the photorespiration in the IspS and MEP*-IspS strains. A previous study had shown that inactivation of the key genes of the 2-PG cycle, *glcD1* and *glcD2*, encoding glycolate dehydrogenase, completely impaired photorespiration in *Synechocystis* but resulted in an HCR phonotype (Eisenhut et al. 2008). The *glcD1* and *glcD2* were, therefore, inactivated in the *Synechocystis* wild-type (WT) strain, as well as the IspS and MEP*-IspS mutant strains. The resulting mutants in which the *glcD1* gene was replaced with the erythromycin resistance cassette Em^r^ and the *glcD2* gene was replaced with the spectinomycin resistance cassette Spec^r^ were designated as ΔglcD1/D2, IspSΔglcD1/D2, and MEP*-IspSΔglcD1/D2, respectively (Fig. 2a). Complete segregation and correct gene insertions were verified by PCR and sequencing (Fig. 2b–d).

We then investigated whether the wild-type (WT) and the mutant strains ΔglcD1/D2, IspSΔglcD1/D2, and MEP*-IspSΔglcD1/D2 would exhibit the HCR phenotype. As shown in Fig. 3b, the strain ΔglcD1/D2 did not grow under normal conditions and required the supplementation of additional inorganic carbon. By contrast, strains IspSΔglcD1/D2 and MEP*-IspSΔglcD1/D2 grew as fine as that of the WT. This demonstrates that introducing an additional energy consuming pathway can indeed make photorespiration dispensable, and the mutant can grow under the ambient conditions.

Although strains IspSΔglcD1/D2 and MEP*-IspSΔglcD1/D2 did not grow better than the WT (Additional file 1: Fig. S1), the isoprene production of strains IspSΔglcD1/D2 (130.64 ± 11.26 μg L^−1^) and MEP*-IspSΔglcD1/D2 (184.18 ± 8.68 μg L^−1^) were approximately twofold higher than that of the strains IspS and MEP*-IspS, respectively (Figs. 2a, 3a). The isoprene productivity of strain MEP*-IspSΔglcD1/D2 newly developed in this work was approximately 2.3 µg L^−1^ h^−1^, which was lower than the highest isoprene productivity in *Synechocystis* (12.8 µg L^−1^ h^−1^) reported previously (Chaves et al. 2016). Furthermore, the isoprene production rate to biomass was analyzed. On the sixth day, the cells density (OD_730_) reached 2.6 (Additional file 1: Fig. S1), which is about 0.96 g dry cell weight L^−1^ according to a predetermined correlation factor of 0369 g L^−1^ per OD_730_ in a previous report (Gao et al. 2016). Therefore, the isoprene production rate to biomass of strain MEP*-IspSΔglcD1/D2 was about 0.19 mg g^−1^ and previously reported the highest one was 0.73 mg g^−1^, indicating driving more carbon flux to isoprene from biomass will greatly contribute to isoprene production in *Synchocystis* (Chaves et al. 2016). This suggests that the photochemical energy consumed by photorespiration could be redirected towards isoprene synthesis.

### Glycolate is accumulated and secreted extracellularly in photorespiration-impaired mutants

Isoprene biosynthesis can rescue the HCR phenotype of impairing photorespiration (Fig. 3b). This indicates that the isoprene biosynthesis in strains IspSΔglcD1/D2 and MEP*-IspSΔglcD1/D2 can completely consume the excess ATP and NADPH that are otherwise consumed by photorespiration.

To determine the fate of photorespiratory glycolate after impairing the photorespiratory glycolate metabolism, the intracellular and extracellular concentration of glycolate of the WT and all mutant strains were determined and comparatively analyzed (Fig. 3c). Figure 3c showed that trace amount of intracellular glycolate accumulation (< 1.5 µM) was observed in all strains tested. Extracellular glycolate was at undetectable level in strains WT, IspS, and MEP*-IspS. However, large amount of extracellular glycolate was detected in photorespiration-impaired mutants IspSΔglcD1/D2, MEP*-IspSΔglcD1/D2, and ΔglcD1/D2. Moreover, the extracellular glycolate concentration of isoprene-producing strains IspSΔglcD1/D2 and MEP*-IspSΔglcD1/D2 was 40% lower than that of the ΔglcD1/D2 strain, indicating that the introduced energy consuming isoprene synthetic pathway can reduce photorespiration, but cannot avoid the oxygentation of Rubisco.

When the photorespiratory pathway was impaired upon knocking out of *glcD1/2*, glycolate was accumulated and the energy for glycolate metabolism might be saved and used for other biochemical process, such as isoprene biosynthesis. To further confirm if the energy saved from blocking glycolate metabolism can contribute to isoprene production, stoichiometric analysis was conducted. Based on the stoichiometry formula containing ATP and reducing equivalents (Additional file 1: Table S3), the ATP and NAD(P)H amounts containing in isoprene biosynthesis and glycolate metabolism were calculated and analyzed (Additional file 1: Table S4).

As shown in Additional file 1: Table S4, upon blocking the photorespiratory cycle, isoprene synthesis could consume a large part of the energy that otherwise is associated with glycolate metabolism. The stronger the isoprene pathway was, the higher the energy consumption ratio reached. The energy calculation also showed that there was a negative correlation between the flux of isoprene synthesis and photorespiratory cycle, which is consistent with what was shown in the Fig. 3c. These data further indicated that the photosynthetic conversion of CO_2_ to isoprene can consume the energy saved from impaired photorespiration and the impaired photorespiration can contribute isoprene production.

It is interesting that the isoprene-producing and photorespiration-impaired mutant strains still produced considerable amount of glycolate but did not exhibit the HCR phenotype, nor did the glycolate produce affect cell growth. This further demonstrated that glycolate metabolism is not essential, nor is glycolate toxic, for cyanobacteria. These data also support our hypothesis that the primary physiological role of photorespiration is to dissipate excess energy rather than detoxifying 2-PG, as photorespiration can be impaired once an alternative energy consumption pathway is available.

To further determine whether the extracellularly secreted glycolate could be metabolized via an unknown pathway, 1 mM ^13^C-labeled glycolate was added to the cultures of strains WT, IspS and MEP*-IspS grown for 48 h, and then cultivated for another 48 h. The initial and final extracellular concentration of ^13^C-labeled glycolate were analyzed by LC–MS (Additional file 1: Fig. S2). The data showed that no significant change of the extracellular concentration of ^13^C-labeled glycolate was observed in all three strains. Photosynthetic microorganisms can only take up and metabolize very little extracellular glycolate (Hess and Tolbert 1967), our result, thus, demonstrated isoprene synthesis did not drive the uptake of the extracellularly supplemented glycolate. This also suggests the extracellularly secreted glycolate in *glcD1*/*glcD2* disrupting strains cannot be re-assimilated either. Furthermore, no significant change of 3-P-glycerate (Additional file 1: Table S5) and not detectable pyruvate of these strains indicated that isoprene synthesis did not drive the metabolism of intracellular glycolate either.

The secretion of the accumulated glycolate was also observed in a previous study, where an inhibitor was supplemented to impair the oxidation of glycolate in cyanobacteria (Norman and Colman 1988). In that study, the extracellular concentration of glycolate can reach a level 20-fold that of the intercellular concentration of glycolate, when incubated with 100% O_2_ which enhanced photorespiration (Norman and Colman 1988). Therefore, our data, together with this previous study (Norman and Colman 1988), demonstrated that impairing glycolate oxidation would result in glycolate accumulation. The fact that the excess glycolate was secreted extracellularly indicates the photorespiratory metabolism of glycolate is not essential for cyanobacteria, so metabolizing the glycolate should not be considered as the primary physiological function of photorespiration in cyanobacteria.

### Improved photosynthetic performance in IspS and MEP*-IspS strains

**Fig. 4:**
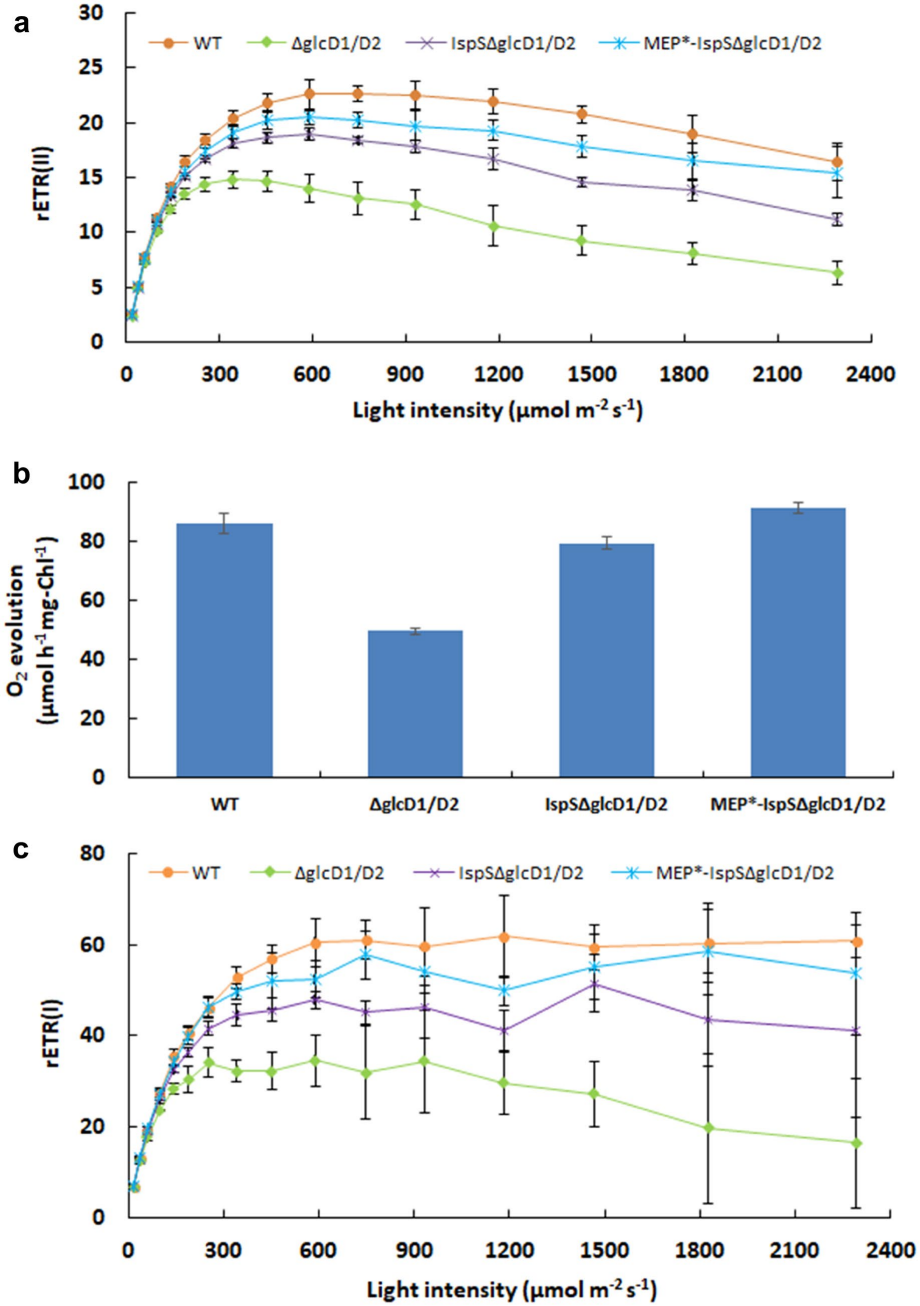
Analysis of PSII and PSI activity. **a** Light response curve of rETR(II), the relative electron transport rate of PSII. **b** O_2_ evolution rate under 300 μmol photons m^−2^ s^−1^. **c** Light response curve of rETR(I), the relative electron transport rate of PSI. The cells grown under HC and measurements were done at LC conditions. Error bars indicate standard deviations (SD) of the data from three independent experiments. For each experiment, three technical replicates were performed

To investigate the effect of impairing photorespiration and/or introducing the energy consuming isoprene synthetic pathway on the activity of PSII, chlorophyll fluorescence kinetics were investigated. The light response curve of rETR(II), the relative electron transport rate of PSII (Fig. 4a) and *Y*(II), and the effective quantum yield of PSII (Additional file 1: Fig. S3a) were measured under light intensities ranging from 18 to 2292 μmol photons m^−2^ s^−1^.

Under low light intensities (18–37 μmol m^−2^ s^−1^), there were almost no difference in rETR(II) and *Y*(II) among all strains (Fig. 4a; Additional file 1: Figs. S3a, S4a, S5a, S6a, b, and S7a, b). That means impairing glycolate metabolism did not result in decrease of photosynthetic activity of PSII at low light intensities. As no photoinhibition occurs at low light intensities, there is no need to initiate photorespiration to dissipate excess energy; therefore, impairing photorespiration does not affect photosynthesis.

When light intensity was increased from 60 to 2292 μmol photons m^−2^ s^−1^, the rETR(II) (Fig. 4a; Additional file 1: Figs. S4b, S6a, S7a) and *Y*(II) (Additional file 1: Figs. S3a, S5b, S6b, S7b) of strains ΔglcD1/D2, IspSΔglcD1/D2, and MEP*-IspSΔglcD1/D2 exhibited decreases of the photochemical activity of PSII ranging from 5.6% to 60%, 2.2% to 30%, and 0.45% to 13% (Table 1), respectively. This means that impairing photorespiration resulted in a more severe photoinhibition of PSII along with increasing light intensity, while the introduced and optimized isoprene-synthetic pathway was able to recover approximately 60% and 80% of the photoinhibition of PSII, respectively. The PSII photoinhibition recovery in strain MEP*-IspSΔglcD1/D2 was approximately 1.3-fold higher than that of the strain IspSΔglcD1/D2, indicating that further enhancing the flux of the introduced isoprene synthetic pathway might potentially completely recover the photoinhibition of PSII under higher light intensities.

**Table 1:** Analysis of PSII and PSI activity decrease in the photorespiration-impaired mutants compared to the WT

Light intensity	Parameters	ΔglcD1/D2 (%)	IspS	MEP*-IspS
			ΔglcD1/D2 (%)	ΔglcD1/D2 (%)
Normal	rETR(II) and *Y*(II)	5.6–10	2.2–4.2	0.45–2.2
	rETR(I) and *Y*(I)	0–14	− 1.47 to 3.8	− 2.6 to 1.4
High	rETR(II) and *Y*(II)	14–60	5.5–30	3–13
	rETR(I) and *Y*(I)	20–73	8.1–32	2.7–11.6

Normal light intensity, 60–100 μmol m^−2^ s^−1^

High light intensity, 142–2292 μmol m^−2^ s^−1^

Moreover, the introduced isoprene synthetic pathway was able to recover the decrease of the saturation light point that resulted from impairing photorespiration. As Fig. 4a shown, the rETR(II) of strainΔglcD1/D2 reached the highest value (approximately 14) under around 300 μmol photons m^−2^ s^−1^, whereas the rETR(II) of the WT kept increasing and reached the highest value of 22 under approximately 600 μmol photons m^−2^ s^−1^, indicating that the impairing of photorespiration resulted in a decrease of the saturation light point from 600 to 300 μmol photons m^−2^ s^−1^. Interestingly, the rETR(II) of strains IspSΔglcD1/D2 and MEP*-IspSΔglcD1/D2 reached their respective highest values (19.0 and 20.5, respectively) under around 600 μmol photons m^−2^ s^−1^, which was similar to the saturation light point of the WT (Fig. 4a).

Furthermore, the oxygen evolution rate, another sensitive indicator of PSII function, was investigated under 300 μmol photons m^−2^ s^−1^, the half-saturation point of the WT. Figure 4b shows that the oxygen evolution rate of strainΔglcD1/D2 was approximately 42% lower than that of the WT (*P* < 0.01). Interestingly, there were no significant differences between the oxygen evolution rates of the strains IspSΔglcD1/D2, MEP*-IspSΔglcD1/D2 and WT, under 300 μmol photons m^−2^ s^−1^. This shows that impairing photorespiration inhibited photosynthetic oxygen evolution, and isoprene synthesis was able to recover the resulting decrease of oxygen evolution.

To further investigate the effects of impairing photorespiration and/or introducing the isoprene synthetic pathway on the energy conversion efficiency of PSI, rETR(I) (Fig. 4c), *Y*(I) (Additional file 1: Fig. S3b) and the decrease of rETR(I) and *Y*(I) compared with the WT (Additional file 1: Fig. S6c, d) were also analyzed (Klughammer and Schreiber 2008). Similar to the effect on PSII, under the low light conditions from 18 to 37 μmol m^−2^ s^−1^, rETR(I) and *Y*(I) were similar, if not identical, in all strains (Fig. 4c; Additional file 1: Figs. S3b, S4c, S5c, S6c, d, S7c, d). That means impairing photorespiration did not result in the decrease of photosynthetic activity of PSI at such low light intensities. The data listed in Table 1 show that the introduced isoprene pathway in the mutants IspSΔglcD1/D2 and MEP*-IspSΔglcD1/D2 was able to completely recover the PSI inhibition under normal light conditions (60 μmol m^−2^ s^−1^ to 100 μmol m^−2^ s^−1^), and recover approximately 60%–90% of the photoinhibition of PSI under light intensities ranging from 142 to 2292 μmol photons m^−2^ s^−1^.

Impairing photorespiration resulted in approximately 34% decrease of PSII activity and 43% decrease of PSI activity at the light-saturation point, indicating that PSI was more sensitive to inhibition generated by impairing photorespiration than PSII (Table 2). The introduced isoprene pathway was able to recover approximately 75% of the photoinhibition of PSII and nearly 98% of the photoinhibition of PSI under 588 μmol photons m^−2^ s^−1^, the light-saturation point of the WT. This indicates that PSI recovers more easily from the photoinhibition resulted from the impairing of photorespiration than PSII does.

**Table 2:** Measurement of chlorophyll fluorescence kinetics for photorespiration-impaired strains and WT under 588 μmol photons m^−2^ s^−1^

Strain	rETR(II)	*Y*(II)	rETR(I)	*Y*(I)	*Y*(ND)	*Y*(NA)
WT	23.63 ± 0.80	0.096 ± 0.004	56.83 ± 3.35	0.37 ± 0.01	0.59 ± 0.01	0.03 ± 0.01
ΔglcD1/D2	15.6 ± 1.35	0.063 ± 0.006	32.6 ± 4.67	0.28 ± 0.05	0.71 ± 0.07	0.01 ± 0.01
IspSΔglcD1/D2	20.03 ± 0.95	0.081 ± 0.004	47.63 ± 5.02	0.33 ± 0.01	0.65 ± 0.02	0.02 ± 0.01
MEP*-IspSΔglcD1/D2	21.63 ± 1.25	0.088 ± 0.005	56.4 ± 7.4	0.35 ± 0.01	0.63 ± 0.02	0.02 ± 0.02

*Y*(II), effective quantum efficiency of PSII

rETR(II), relative electron transport rate of PSII

*Y*(I), effective quantum efficiency of PSI

rETR(I), relative electron transport rate of PSI

*Y*(ND), the quantum yield of non-photochemical energy dissipation due to donor-side limitation

*Y*(NA), the quantum yield of non-photochemical energy dissipation due to acceptor-side limitation

Data represent the means from three independent measurements ± SD. For each experiment, three technical replicates were performed

To further understand the fate of the quantum yield of PSI, the non-photochemical dissipation of the PSI quantum yield: *Y*(ND) (the quantum yield of non-photochemical energy dissipation due to donor-side limitation) and *Y*(NA) (the quantum yield of non-photochemical energy dissipation due to acceptor-side limitation) were analyzed (Zhou et al. 2016) under the high light intensity of 588 μmol photons m^−2^ s^−1^ (Table 2). *Y*(ND) of the photorespiration-impaired strain ΔglcD1/D2 increased 20%, while *Y*(NA) of ΔglcD1/D2 decreased 6.7% compared to the *Y*(ND) and *Y*(NA) of the WT, respectively. This result indicates that the donor-side limitation is the reason for the photoinhibition of PSI, rather than the acceptor side. This further indicates that the excess photochemical energy from the photosystems, rather than the oxygen-induced inhibition of carbon fixation, causes the HCR phenotype when photorespiration is impaired.

## Conclusions

In summary, the production of isoprene in photorespiration-impaired strain doubled, indicating the energy consumed by photorespiration was reused for isoprene production. We further demonstrated that the introduced isoprene synthetic pathway can recover the photoinhibition of both PSII and PSI that normally results from impairing of photorespiration, thus providing an alternative strategy for avoiding or engineering photorespiration (Kebeish et al. 2007; Shih et al. 2014) in photosynthetic organisms. By designing and introducing an energy consuming pathway, we can, therefore, make the photorespiration of cyanobacteria dispensable, and put the otherwise consumed energy into the photosynthetic conversion of CO_2_ to useful chemicals.

### Supplementary Information

**Additional file 1: Figure S1.** Growth profile of strains during measurement of isoprene production in sealed bottles with 50 mM NaHCO_3_ supplement. **Figure S2.** The relative percentage of extracellular concentration of ^13^C-labeled glycolate in WT, IspS and MEP*-IspS strains detected by LC–MS. Initial concentration was detected right after adding 1 mM ^13^C-labeled glycolate to the cultures. Final concentration was detected after strains were cultivated sequentially for 2 days. **Figure S3.** Light Intensity response curve of Y(II) (a) and Y(I) (b) of all strains. Y(II), effective quantum efficiency of PS II; Y(I), effective quantum efficiency of PS I. Error bars indicate standard deviation (SD) of the data from three independent experiments. For each experiment, three technical replicates were performed. **Figure S4.** The enlarged figure of Fig. 4a, c at the range of 18–37 (a, c) and 60–100 μmol m^−2^ s^−1^ (b, d). **Figure S5.** The enlarged figure of Fig. S3 at the range of 18–37 (a, c) and 60–100 μmol m^−2^ s^−1^ (b, d). **Figure S6.** Comparable analysis of the photochemical efficiency decrease of all strains corresponding light intensity. (a) rETR(II), elative electron transport rate of PSII. (b) Y(II), effective quantum efficiency of PSII. (c) rETR(I), relative electron transport rate of PSI. (d) Y(I), effective quantum efficiency of PSI. **Figure S7.** The enlarged figure of Fig. S6 at the range of 18–100 μmol m^−2^ s^−1^. **Table S1.** Strains and plasmids used in this study. **Table S2.** Primers used in this study. **Table S3.** Stoichiometry formula containing ATP and reducing equivalents for isoprene biosynthesis from photosynthesis and photorespiration. **Table S4.** The amounts of ATP and NAD(P)H containing in isoprene biosynthesis and glycolate re-assimilation. **Table S5.** The intracellular amounts of 3-P-glycerate in WT and mutant strains.

## Data Availability

The data and the materials are all available in this article as well as the Additional file.

## Abbreviations

2-PG: 2-Phosphoglycolate
Rubisco: Ribulose-1,5-bisphosphate carboxylase/oxygenase
HCR: High-CO_2_-requiring
CCM: CO_2_-concentrating mechanism
*Synechocystis*: *Synechocystis* sp. PCC 6803
IspS: Isoprene synthase
*Y*(II): The effective quantum yield of PSII
rETR(II): The relative electron transport rate of PSII
*Y*(I): The effective quantum yield of PSI
rETR(I): The relative electron transport rate of PSI
*Y*(ND): The quantum yield of non-photochemical energy dissipation due to donor-side limitation
*Y*(NA): The quantum yield of non-photochemical energy dissipation due to acceptor-side limitation
RuBP: Ribulose biphosphate
3-PGA: Glycerate-3-phosphate
G3P: Glyceraldehyde-3-phosphate
MEP: Methylerythritol phosphate
DXP: 1-Deoxy-d-xylulose-5-phosphate
DXS: DXP synthase
DXR: DXP reductoisomerase
CDP-ME: Diphosphocytidylyl methylerythritol
CDP-MEP: Diphosphocytidylyl methylerythritol 2-phosphate
MEcPP: Methylerythritol 2,4-cyclodiphosphate
HMBPP: Hydroxymethylbutenyl 4-diphosphate
IPP: Isopentenyl pyrophosphate
DMAPP: Dimethylallyl pyrophosphate
IspD: 4-Diphosphocytidyl-2C-methyl-d-erythritol synthase
IspE: 4-(Cytidine-5ʹ-diphospho)-2-C-methyl-d-erythritol kinase
IspF: 2C-Methyl-d-erythritol 2,4-cyclodiphosphate synthase
IspG: 4-Hydroxy-3-methylbut-2-enyl-diphosphate synthase
IspH: HMBPP reductase
IDI: IPP isomerase
WT: Wild type
