# Supplementary material for: Impairing photorespiration increases photosynthetic conversion of CO2 to isoprene in engineered cyanobacteria
Source: Bioresour Bioprocess. 2021 May 21;8(1):42. doi: 10.1186/s40643-021-00398-y (PMC10992918; doi:10.1186/s40643-021-00398-y)
Supplement: Supplementary file 1 — Additional file 1: Figure S1. Growth profile of strains during measurement of isoprene production in sealed bottles with 50 mM NaHCO3 supplement. Figure S2. The relative percentage of extracellular concentration of 13C-labeled glycolate in WT, IspS and MEP*-IspS strains detected by LC–MS. Initial concentration was detected right after adding 1 mM 13C-labeled glycolate to the cultures. Final concentration was detected after strains were cultivated sequentially for 2 days. Figure S3. Light Intensity response curve of Y(II) (a) and Y(I) (b) of all strains. Y(II), effective quantum efficiency of PS II; Y(I), effective quantum efficiency of PS I. Error bars indicate standard deviation (SD) of the data from three independent experiments. For each experiment, three technical replicates were performed. Figure S4. The enlarged figure of Fig. 4a, c at the range of 18–37 (a, c) and 60–100 μmol m−2 s−1 (b, d). Figure S5. The enlarged figure of Fig. S3 at the range of 18–37 (a, c) and 60–100 μmol m−2 s−1 (b, d). Figure S6. Comparable analysis of the photochemical efficiency decrease of all strains corresponding light intensity. (a) rETR(II), elative electron transport rate of PSII. (b) Y(II), effective quantum efficiency of PSII. (c) rETR(I), relative electron transport rate of PSI. (d) Y(I), effective quantum efficiency of PSI. Figure S7. The enlarged figure of Fig. S6 at the range of 18–100 μmol m−2 s−1. Table S1. Strains and plasmids used in this study. Table S2. Primers used in this study. Table S3. Stoichiometry formula containing ATP and reducing equivalents for isoprene biosynthesis from photosynthesis and photorespiration. Table S4. The amounts of ATP and NAD(P)H containing in isoprene biosynthesis and glycolate re-assimilation. Table S5. The intracellular amounts of 3-P-glycerate in WT and mutant strains. [file 40643_2021_398_MOESM1_ESM.docx]

**Impairing photorespiration increases photosynthetic conversion of CO_2_ to isoprene in engineered cyanobacteria**

Jie Zhou†^d^, Fan Yang†^a, b^, Fuliang Zhang†^a, b^, Hengkai Meng^a, c^, Yanping Zhang^a^, Yin Li*^a^


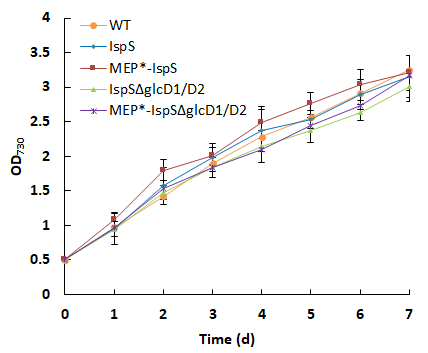


**Figure S1** Growth profile of strains during measurement of isoprene production in sealed bottles with 50 mM NaHCO_3_ supplement.

**Figure S2** The relative percentage of extracellular concentration of ^13^C-labelled glycolate in WT, IspS and MEP^*^-IspS strains detected by LCMS. Initial concentration was detected right after adding 1 mM ^13^C-labelled glycolate to the cultures. Final concentration was detected after strains were cultivated sequentially for 2 days.

**
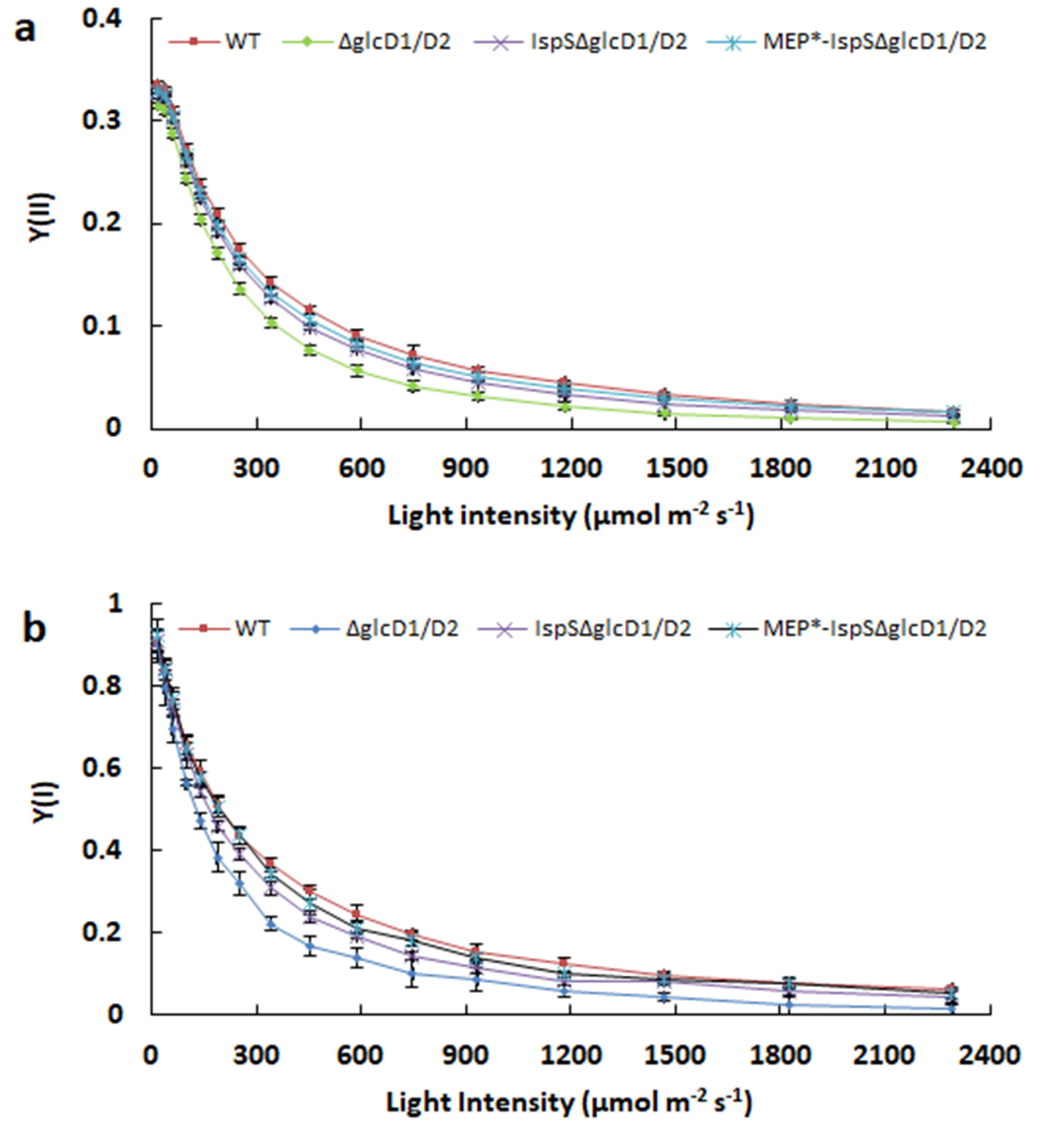
**

**Figure S3** Light Intensity response curve of Y(II) (a) and Y(I) (b) of all strains. Y(II), effective quantum efficiency of PS II; Y(I), effective quantum efficiency of PS I. Error bars indicate standard deviation (SD) of the data from three independent experiments. For each experiment, three technical replicates were performed.

**
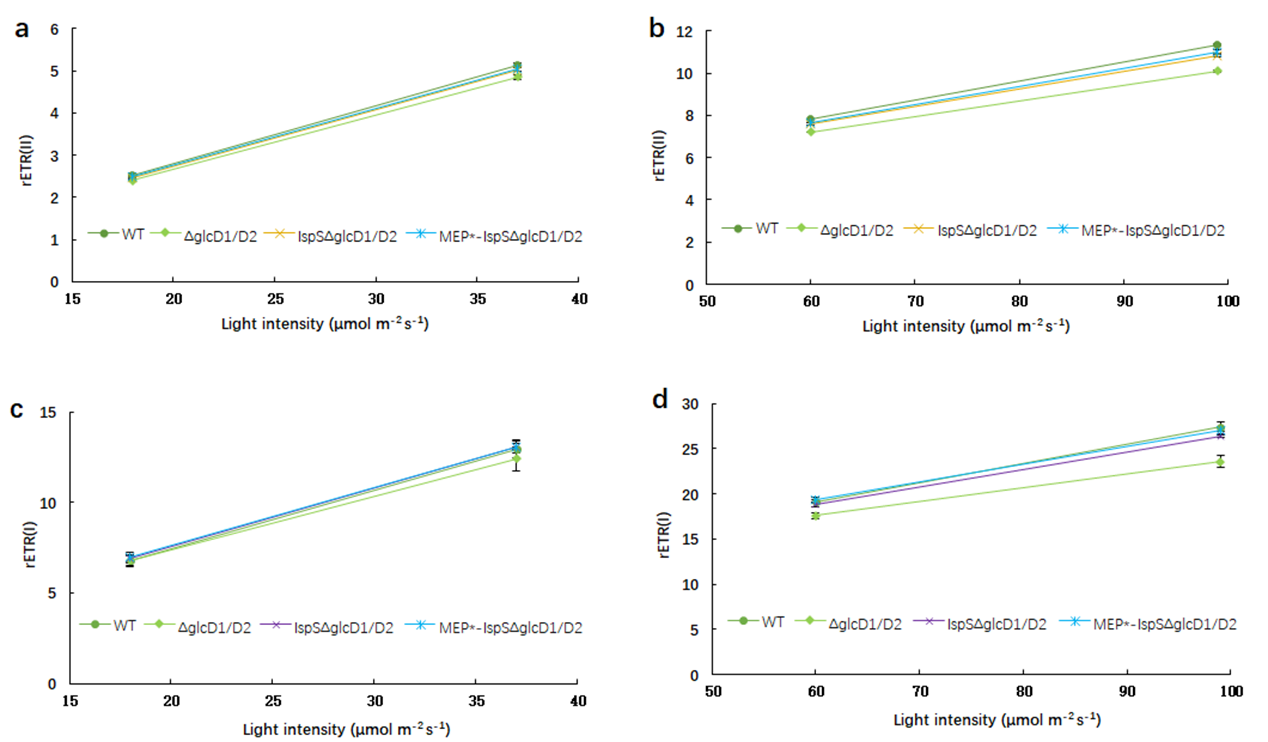
**

**Figure S4** The enlarged figure of Fig. 4a & c at the range of 18-37 (a, c) and 60-100 μmol m^-2^ s^-1^ (b, d).

**
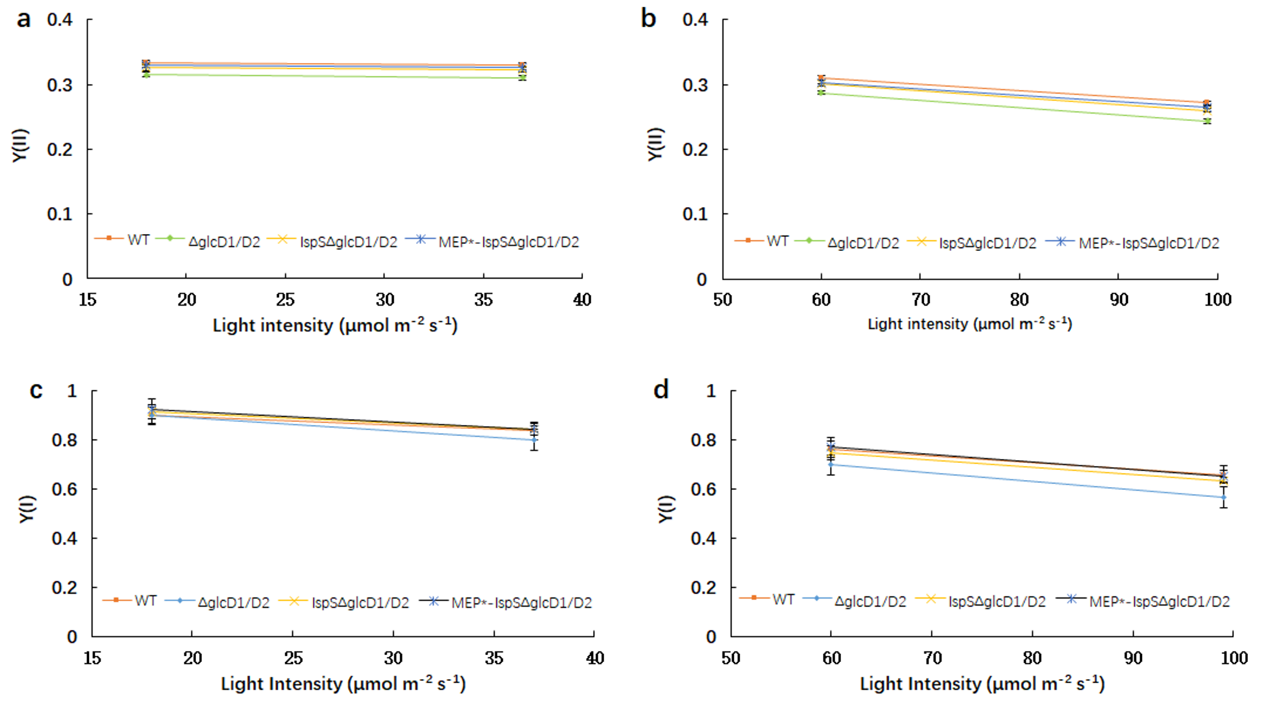
**

**Figure S5** The enlarged figure of Fig. S3 at the range of 18-37 (a, c) and 60-100 μmol m^-2^ s^-1^ (b, d).

**
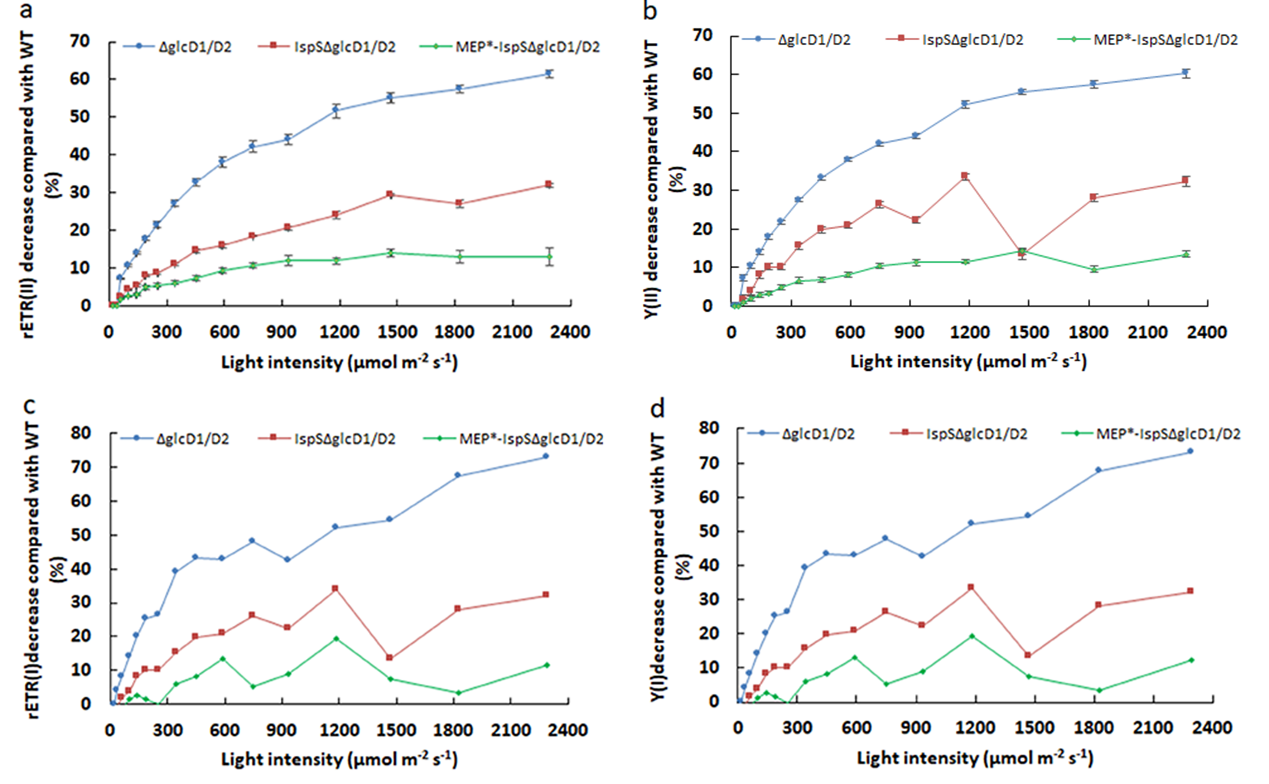
**

**Figure S6** Comparable analysis of the photochemical efficiency decrease of all strains corresponding light intensity. (a) rETR(II), elative electron transport rate of PSII. (b) Y(II), effective quantum efficiency of PSII. (c) rETR(I), relative electron transport rate of PSI. (d) Y(I), effective quantum efficiency of PSI.

**
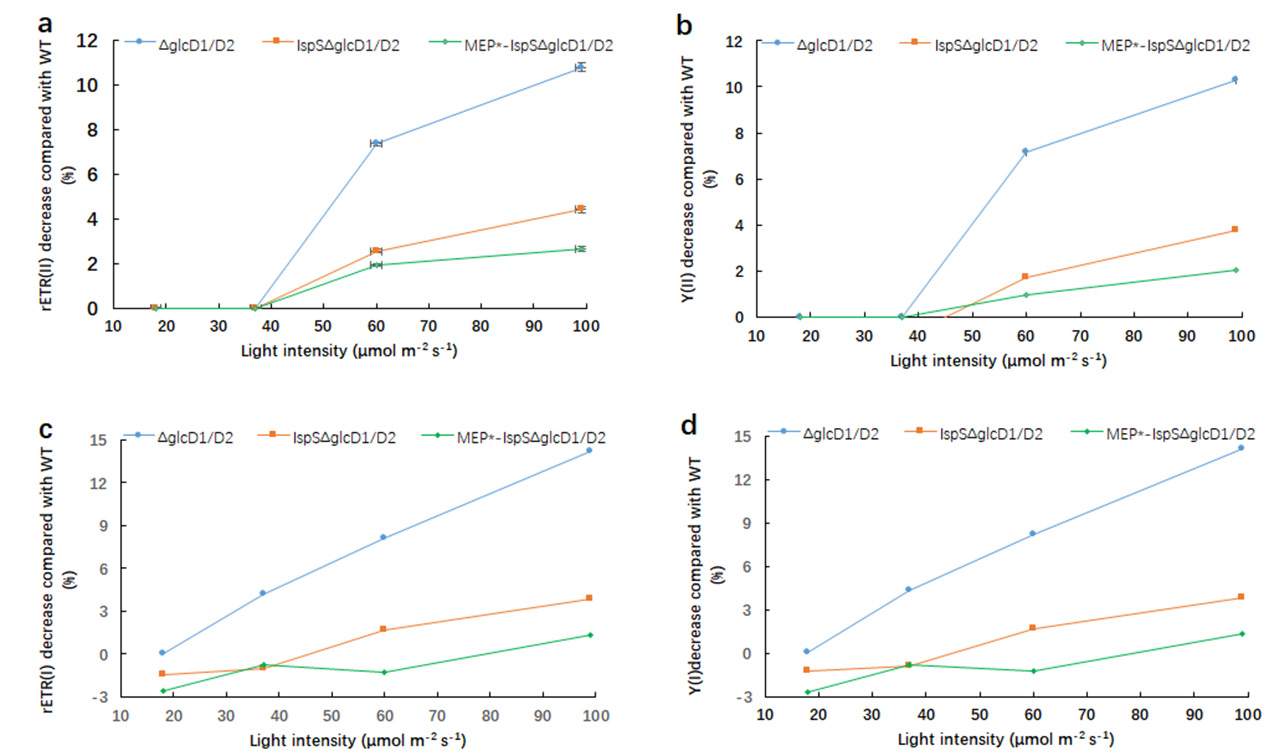
**

**Figure S7** The enlarged figure of Fig. S6 at the range of 18-100 μmol m^-2^ s^-1^.

**Table S1** Strains and plasmids used in this study.

| Strains and plasmids | Relevant characteristics | Reference |
| --- | --- | --- |
| **Strains** |  |  |
| *E. coli* DH5α | Commercial strain for plasmid construction | Lab storage |
| *Synechocystis* PCC6803 | Wild type | Lab storage |
| IspS | pta::Pcpc-*isp*S, Km^r^ | This work |
| MEP*-ispS | phaCE:: Pcpc-*dxs-idi-ispD*-*ispF*, Cm^r^;  pta::Pcpc-*ispS*, Km^r^ | This work |
| ΔglcD1/D2 | *glc*D1::Em^r^; *glc*D2::Spec^r^ | This work |
| ispSΔglcD1/D2 | pta::Pcpc-*isp*S, Km^r^  *glc*D1::Em^r^; *glc*D2::Spec^r^ | This work |
| MEP*-ispSΔglcD1/D2 | phaCE:: Pcpc-*dxs-idi-isp*D-*isp*F, Cm^r^;  pta::Pcpc-*isp*S, Km^r^  *glc*D1::Em^r^; *glc*D2::Spec^r^ | This work |
| **Plasmids** |  |  |
| pSM1 | pMD18-Tderivate, Amp^r^ Cm^r^, containing phaCE knockout cassette | Zhou et al, 2012 |
| pSM2 | pMD18-Tderivate, Amp^r^ Km^r^, containing pta knockout cassette | Zhou et al, 2012 |
| pSM2-ispS | pSM2 derivate, containing Pcpc-*isp*S expression cassette | This work |
| pSM1-MEP* | pSM1 derivate, containing Pcpc-*dxs-idi-isp*D-*isp*F expression cassette | This work |
| pEasy-blunt-simple | Commercial cloning vector, Amp^r^ Km^r^ | TransGen |
| pEasy-ΔglcD1 | pEasy-blunt-simple derivate, Amp^r^ Km^r^ Em^r^ containing *glc*D1knockout cassette | This work |
| pEasy-ΔglcD2 | pEasy-blunt-simple derivate, Amp^r^ Km^r^ Spec^r^ containing *glc*D2 knockout cassette | This work |

**Table S2** Primers used in this study.

| Primers | Sequence (5’-3’) |
| --- | --- |
| Pcpc560-F | acctgtagagaagagtccctg |
| Pcpc560-R | tgaattaatctcctacttgac |
| ispS-F | atgatctgtgctacctcctcc |
| ispS-R | ttaaacgtacattaactggttg |
| dxs-F | atgagttttgatattgccaaatacc |
| dxs-R | ttatgccagccaggccttgatt |
| idi-F | atgagttttgatattgccaaatacc |
| idi-R | ttatttaagctgggtaaatgc |
| ispD-F | atggcaaccactcatttggatg |
| ispD-R | ttatgtattctcctgatggatgg |
| ispF-F | atgcgaattggacacggttttgacg |
| ispF-R | tcattttgttgccttaatgagtagcg |
| glcD1-Up-F | tcaataaatttcctccattttcttgtcc |
| glcD1-Up-R | caccgcccaatccgtggccgatatcactcgagttgcggcgggcatcgtcccagcgggc |
| glcD1-Down-F | gcccgctgggacgatgcccgccgcaactcgagtgatatcggccacggattgggcggtg |
| glcD1-Down-R | tggccattttctcccccgtcaacgc |
| Em-F | cagtctcgaggaccaaaagtataaaacctttaag |
| Em-R | cagtctcgagttacttattaaataatttatagc |
| glcD2-Up-F | cccatcgaagtggaaatgcccactgg |
| glcD2-Up-R | atgaactcgattttataattccttgctaagtctg |
| glcD2-Down-F | cggcaaataatccagttatttttaccaaaaaaaattg |
| glcD2-Down-R | actctggtttaatttcgctccctc |
| Spec-F | tcgagttcatgtgcagctccatc |
| Spec-R | ttatttgccgactaccttggtgatc |

**Table S3** Stoichiometry formula containing ATP and reducing equivalents for isoprene biosynthesis from photosynthesis and photorespiration.

| Processes | Stoichiometry formula | Required ATP (mol/mol) | Required NAD(P)H  (mol/mol) | ^*^Total ATP eq. (mol/mol) |
| --- | --- | --- | --- | --- |
| CBB cycle | 3 CO_2_+ 9 ATP + 6 NADPH + 6 H^+^ → G3P + 9 ADP + 6 NADP^+^ | 9 | 6 | 24 |
| Isoprene from photosynthesis | G3P + 2 ADP + NAD^+^ → pyruvate + 2 ATP + NADH + H^+^ | / | / | / |
|  | Pyruvate + G3P + 3 NAD(P)H + ATP + CTP → isoprene + CO_2_ + ADP + CMP | / | / | / |
|  | 2 ATP + CMP → 2 ADP + CTP | / | / | / |
|  | In total:  5 CO_2_+ 19 ATP + 14 NAD(P)H +14 H^+^→ isoprene + 19 ADP + 14 NAD(P)^+^ | 19 | 14 | 54 |
| Photorespiration in cyanobacteria  ([Bauwe et al., 2010](#_ENREF_1)) | RuBP + O2 → 3-PGA + 2-PG | / | / | / |
|  | 2-PG → glycolate + Pi | / | / | / |
|  | Glycolate + NAD(P)^+^→ glyoxylate + NAD(P)H + H^+^ | / | / | / |
|  | 2 glyoxylate → hydroxypyruvate + CO_2_  Hydroxypyruvate + NAD(P)H + H^+^ → glycerate+ NAD(P)^+^ | / | / | / |
|  | Glycerate + ATP →3-PGA + ADP | / | / | / |
|  | 3-PGA + ATP + NADPH + H^+^→G3P + ADP + NADP^+^ + Pi | / | / | / |
|  | In total,  glycolate + ATP → 0.5 G3P + 0.5 CO_2_ + 0.5 ADP + 0.5 Pi | 1 | 0 | 1 |

*Total ATP equivalent was calculated by assuming 2.5 mol ATP per NAD(P)H ([Peterhansel et al., 2010](#_ENREF_2)). CBB, Calvin Benson Bassham.

“/*”* means not shown.

Bauwe, H., Hagemann, M. and Fernie, A.R. (2010) Photorespiration: players, partners and origin. *Trends Plant Sci* **15**, 330-336.

Peterhansel, C., Horst, I., Niessen, M., Blume, C., Kebeish, R., Kürkcüoglu, S. and Kreuzaler, F. (2010) Photorespiration. *Arabidopsis Book* **8**, e0130-e0130.

**Table S4** The amounts of ATP and NAD(P)H containing in isoprene biosynthesis and glycolate re-assimilation.

| **Strains**  **Energy consumption** | | **WT** | **ΔglcD1/D2** | **IspS** | **IspS**  **ΔglcD1/D2** | **MEP*-IspS** | **MEP*-IspS**  **ΔglcD1/D2** |
| --- | --- | --- | --- | --- | --- | --- | --- |
| Isoprene  biosynthesis | ATP | 0 | 0 | 12.71 | 28.48 | 19.67 | 40.76 |
|  | NAD(P)H | 0 | 0 | 9.36 | 20.98 | 14.49 | 30.03 |
| Glycolate  metabolism | ATP | *296.01 | 0 | *216.38 | 0 | *171.35 | 0 |
|  | NAD(P)H | 0 | 0 | 0 | 0 | 0 | 0 |
| **All energy converted to ATP | Isoprene  biosynthesis | 0 | 0 | 36.12 | 80.94 | 55.91 | 115.83 |
|  | Glycolate  metabolism | *296.01 | 0 | *216.38 | 0 | *171.35 | 0 |

*In strains without *glcD1*/*glcD2* knockout, glycolate was metabolized and recycled, and the amount of glycolate was unknown. Therefore, the amount of glycolate accumulated in strains without *glcD1*/*glcD2* knockout was calculated according to its corresponding *glcD1*/*glcD2* knockout strains.

** All energy was converted to ATP by assuming 2.5 mol ATP per NAD(P)H ([Peterhansel et al., 2010](#_ENREF_2)).

**Table S5** The intracellular amounts of 3-P-glycerate in WT and mutant strains

| Strain | GAP (nmol OD_730_^-1^) |
| --- | --- |
| WT | 0.169±0.071 |
| ΔglcD1/D2 | 0.320±0.021 |
| ispSΔglcD1/D2 | 0.309±0.078 |
| MEP*-ispSΔglcD1/D2 | 0.316±0.100 |
